# Overcoming the Limitations of Conventional Orthognathic Surgery: A Novel Approach Using Implate

**DOI:** 10.3390/jcm14145012

**Published:** 2025-07-15

**Authors:** Valerio Ramieri, Laura Viola Pignataro, Tito Matteo Marianetti, Davide Spadoni, Andrea Frosolini, Paolo Gennaro

**Affiliations:** 1 Ortognatica Roma, Via Nomentana 311, 00137 Roma, Italy; valerioramieri@gmail.com (V.R.); titomatteo.marianetti@gmail.com (T.M.M.); davidespadoni03@gmail.com (D.S.); 2 Maxillofacial Surgery Unit, Department of Medical Biotechnologies, Azienda Ospedaliera Universitaria Senese, 53100 Siena, Italy; andreafrosolini@gmail.com (A.F.); paolo.gennaro@unisi.it (P.G.)

**Keywords:** orthognathic surgery, mandibular angle, mandible widening, mandibular height, facial implants

## Abstract

**Introduction**: This manuscript addresses the limitations of traditional orthognathic surgery in achieving both functional and aesthetic correction in patients with Class II malocclusion and severe mandibular retrusion. Current techniques often struggle to simultaneously address mandibular deficiency and inadequate transverse dimension, leading to unsatisfactory outcomes. **Methods**: Seven male patients underwent bimaxillary osteotomy with mandibular advancement. A novel surgical plate, Implate, was used, which was designed to facilitate precise osteotomy and stabilization. Pre-surgical planning included CBCT scanning, 3D modeling, and surgical simulation. Postoperative assessments included clinical examinations, CT and OPT scans. **Results**: Implate successfully addressed the challenges of conventional techniques, minimizing the formation of bony steps and achieving a more harmonious facial profile. The minimally invasive procedure, with careful periosteal and muscle management, contributed to stable outcomes, and no complications were reported. At the 6-month follow-up, OPT analysis showed a mean mandibular width increase of 18.1 ± 6.2 mm and vertical ramus height gains of 6.0 ± 3.1 mm (left) and 5.8 ± 1.7 mm (right). **Conclusions**: According to our preliminary experience, the integration of Implate into surgical practice offers a significant improvement in treating complex Class II malocclusions. By simultaneously correcting mandibular retrusion and width while minimizing complications, Implate provides a superior solution compared to traditional methods. This innovative approach highlights the potential of combining advanced surgical techniques with personalized 3D-printed implants to achieve optimal functional and aesthetic outcomes. Further prospective studies with controls and longer follow-up are needed to validate the efficacy and reproducibility of Implate in wider clinical use.

## 1. Introduction

Many orthodontic patients undergo treatment for Class II malocclusion to improve both occlusal function and facial aesthetics [1]. This malocclusion often results from underlying skeletal and dental discrepancies, such as maxillary excess, mandibular deficiency, or a combination of both involving the maxilla, mandible, and dentoalveolar structures. Treatment strategies for Class II malocclusions vary based on factors such as growth stages, anteroposterior discrepancies, facial aesthetics, airway considerations, and patient compliance. When there is a significant discrepancy between the maxilla and the mandible, camouflage treatments may lead to periodontal complications such as gingival recession in the lower anterior teeth, root resorption, deteriorating facial aesthetics, and occlusal instability [2]. Therefore, for adult patients with severe skeletal Class II malocclusions, a combination of orthodontic treatment and orthognathic surgery is preferred to improve both the bite and the facial profile [3]. In cases of mandibular retrusion, a Bilateral Sagittal Split Osteotomy (BSSO) surgical procedure is often needed to perform forward and downward movements, correct the malocclusion, and achieve a good aesthetic result.

Moreover, counterclockwise (CCW) rotation is often required to achieve the intended occlusal relationship [4]. However, these mandibular movements inevitably result in the formation of a bony step, which could be insufficient for increasing ramus height and would not produce the linear mandibular contour preferred by patients and essential for facial harmony. Furthermore, when transverse widening of the mandible is indicated, with conventional osteotomies techniques, asymmetrical results could occur, failing to achieve adequate transverse dimension [5]. The challenge, therefore, was to obtain functional and stable occlusion while simultaneously delivering an aesthetically satisfactory mandibular profile [6]. To address these issues, meticulous preoperative planning and precise surgical execution are essential [7]. Surgeons must thoroughly assess the risk of bony step formation and adopt strategies to minimize it. With conventional planning methods and classic osteotomies, providing the necessary ramus height or transverse dimension could be challenging. Ramanathan et al. proposed a mandibular midline osteotomy technique to address transverse deficiencies in cases with a normodivergent maxilla, achieving 2–4 mm widening using bioresorbable fixation and virtual planning [8]. While effective for width correction, this approach does not address concurrent vertical deficiencies. Secondary onlay grafts and implants to refine facial contour can be an alternative, but involve multi-step strategies that not only increase operative time and morbidity, but also elevate the risk of relapse, soft tissue distortion, and complications related to muscle detachment and prosthetic adaptation [9]. The need for separate procedures to address each anatomical plane of deficiency reflects a major limitation in current orthognathic protocols and underscores the value of integrated solutions.

To overcome these limitations, we developed Implate, a custom-designed titanium device that integrates osteosynthesis with volumetric enhancement of the mandibular angle. The aim of this technical note is to describe the design, surgical application, and short-term outcomes of Implate in a case series of patients with Class II malocclusion, requiring both functional mandibular advancement and aesthetic contour restoration.

## 2. Materials and Methods

### 2.1. Patient Selection and Surgical Planning

**Figure 1:**
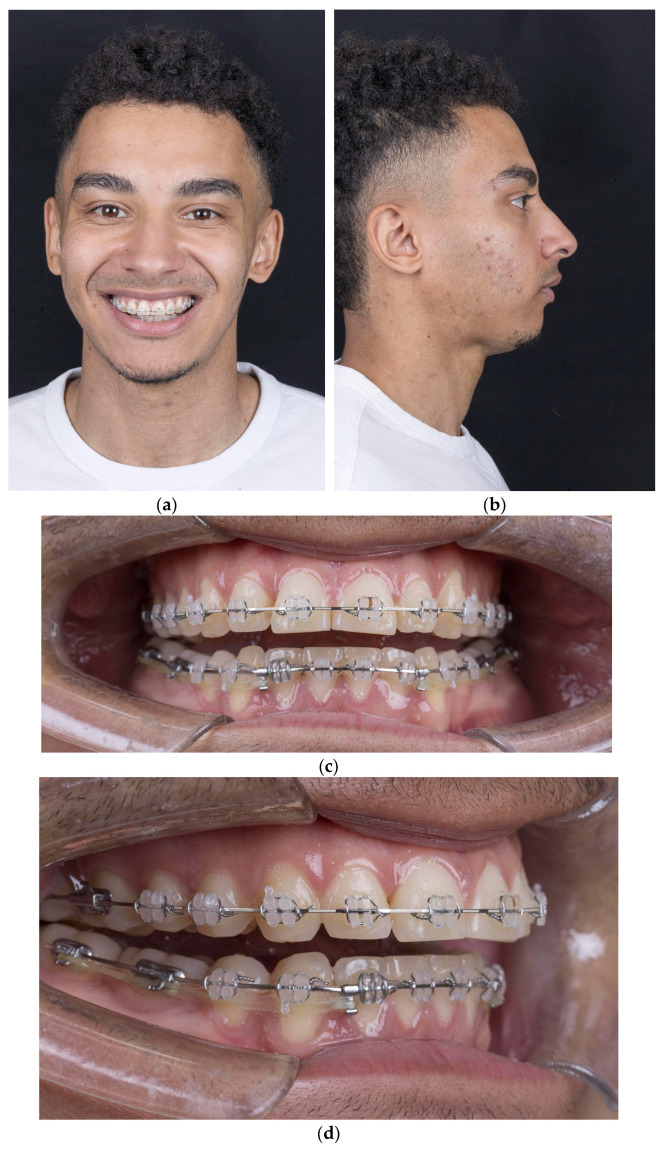
(**a**) Frontal view with smile; (**b**) lateral view showing mandibular retrusion; (**c**) intraoral frontal view with open bite; (**d**) intraoral lateral view.

Patients referred to our orthognathic surgery clinic by their orthodontists between 1 January and 1 March 2025 for the treatment of Class II dentoskeletal malocclusion were considered candidates for the Implate technique. A thorough anamnesis and clinical examination were performed (Figure 1). Once the patients completed the pre-surgical orthodontic treatment and underwent the necessary occlusal decompensation for the surgery, cone-beam computed tomography (CBCT) with a maxillomandibular field of view was performed to confirm the diagnosis and to enable virtual surgical planning.

The scan was transferred to our 3D-planning software (Dolphin Imaging Software, Los Angeles, CA, USA) and the following workflow was applied: CBCT scan segmentation (I); volume orientation (II); creation of a two-dimensional image corresponding to a latero-lateral X-radiograph (III); Rickett’s cephalometric analysis (IV); and surgical planning simulation (V). Decisions regarding the degree of ramus lengthening and angle widening were informed by both individual patient needs and aesthetic targets derived from contemporary morphometric data. In particular, we referenced the ideal gonial morphology reported by Mommaerts et al. (2016), in which a mandibular angle of approximately 125.5° and a pronounced posterior contour were found to correlate with optimal male facial aesthetics [10]. Vertical deficiency was assessed via Ricketts analysis and elongation was planned to optimize lower facial height and support functional occlusal repositioning after CCW rotation. Lateral widening at the mandibular angle was an aesthetic refinement, guided by virtual planning tools and patient-specific simulations. The obtained surgical planning files were shared with an engineering company (Materialise NV, Leuven, Belgium) specializing in Patient-Specific Implant (PSI).

### 2.2. The Implate Project

**Figure 2:**
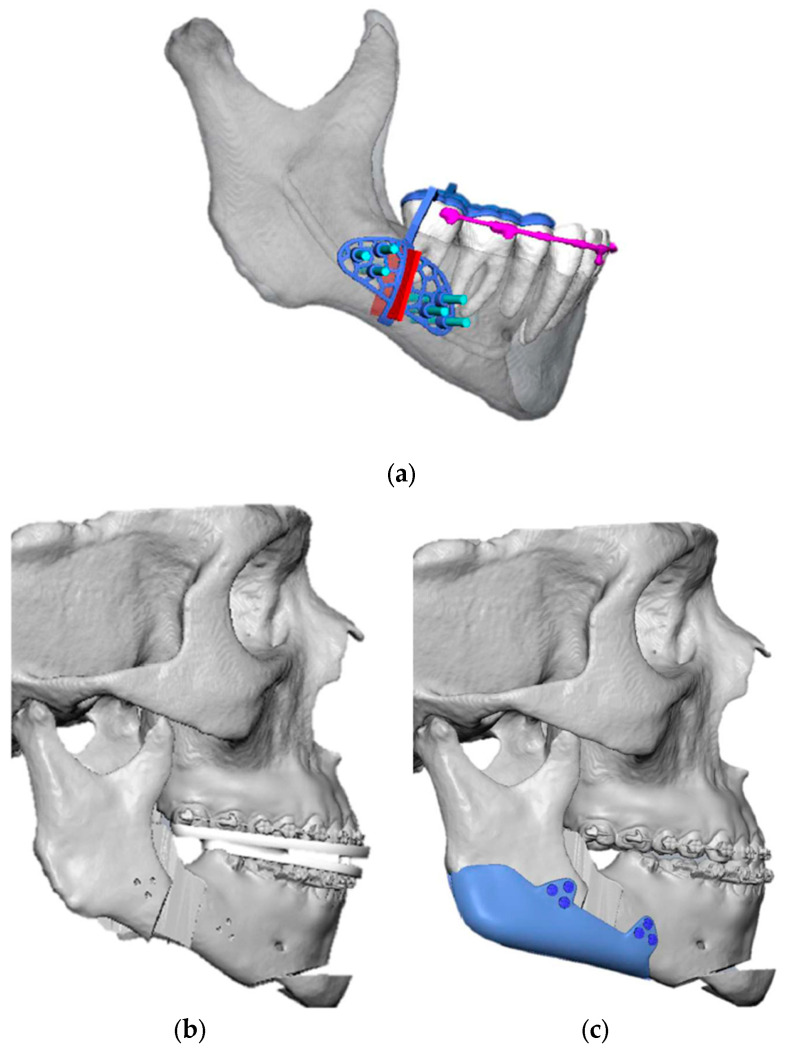
(**a**) VSP of the right mandibular cutting guide; (**b**) VSP of the mandibular reposition; (**c**) VSP of Implate insetting.

**Figure 3:**
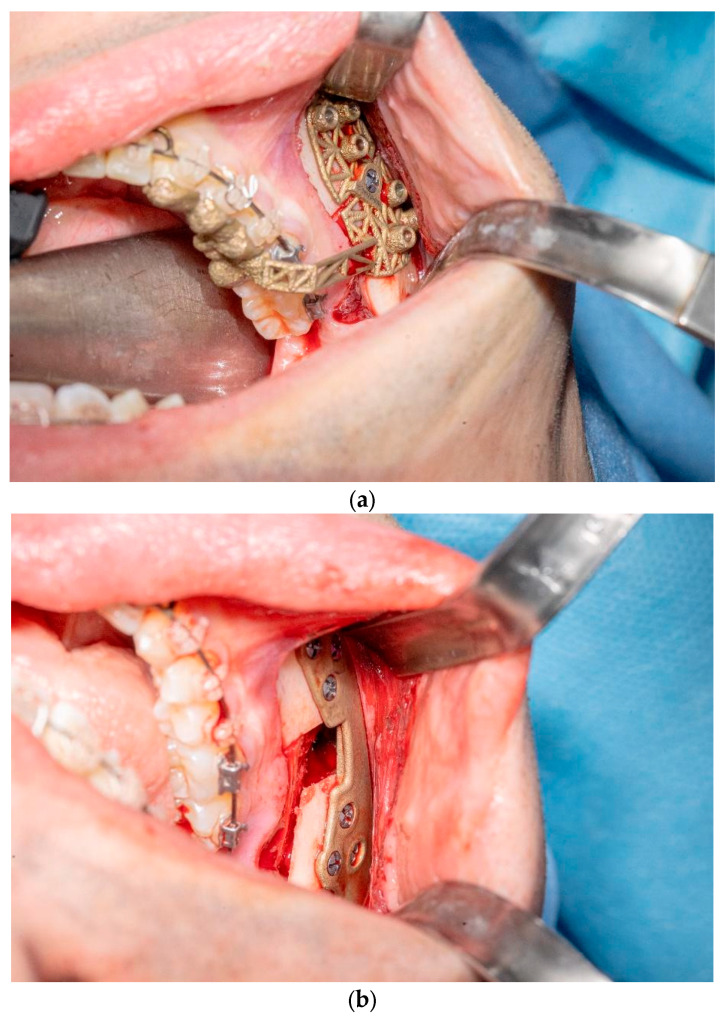
(**a**) Intraoperative insetting of right mandibular cutting guide; (**b**) Implate insetting.

The Implate was designed to wrap around the lower border of the mandible and the posterior margin of the ramus, thereby achieving a linear facial profile symmetrical to the midline (Figure 2). Upon confirmation of the planning, Implate was fabricated from titanium and sterilized in accordance with the company’s protocols. The Implate was manufactured from medical-grade titanium alloy with a flat, non-meshed surface. This choice was based on evidence that smooth surfaces reduce bacterial adhesion and biofilm formation, with up to 80–90% less biofilm formation on flat titanium compared to porous coatings, while maintaining osteogenic cell compatibility [11]. A patient-specific mandibular cutting guide was developed for each case to ensure precise execution of the BSSO. The guide was designed to conform to the outer cortical surface of the mandibular body and ramus, as well as the posterior dental facets, allowing passive intraoperative positioning based on anatomical landmarks (Figure 3a). Temporary fixation holes were incorporated to stabilize the guide during osteotomy. A dedicated lateral groove along the planned osteotomy line accommodated piezoelectric and oscillating surgical instruments, enabling a controlled and reproducible cut. Osteosynthesis was then carried out using predrilled holes aligned with the implant design, ensuring accurate fixation (Figure 2c).

### 2.3. Surgical Procedure

In order to correct the malocclusion, the treatment plan for all seven patients required bimaxillary osteotomies with mandibular advancement. The patients underwent the surgical procedure under general anesthesia: a paramarginal vestibular mandibular incision was made with an extension sufficient to allow for the insertion of the device; following the detachment of the periosteum, we cut the pterigomasseteric sling and the sphenomandibular ligament insertion to reduce the risk of relapse [12]. Later, we created guides for the mandibular osteotomies using a piezoletric device and completed the mandibular split with chisels. Once the bilateral mandibular osteotomies had been performed, occlusion was achieved as pre-surgically planned. Implate was then positioned at the mandibular angles as designed, ensuring osteosynthesis with six titanium screws (three of these anteriorly and three posteriorly) (Figure 3b). The patients were discharged on the first postoperative day with mild edema. No wound dehiscence, hematoma collection, or signs of infection were observed. Patients attended check-ups at 1, 2, and 4 weeks; no postoperative complications or occlusal changes were detected. Orthodontic elastics were not required in any case, and no IMF was necessary. One month after the surgery, once the edema had been resolved, patients underwent a postoperative CT scan, which proved stable osteosynthesis. The radiological data were then superimposed onto the preplanned data and compared.

### 2.4. Postoperative Analysis

Mandibular width and ramus height were measured on pre- and post-operative orthopantomograms (OPT) at the 6-month follow-up. In this study, ramus height was defined as the vertical distance from the deepest point of the sigmoid notch to the most inferior point of the mandibular angle. Mandibular width was measured as the linear distance between the angles of the mandible on both sides. This measurement protocol was developed based on consistent anatomical landmarks and prior studies describing mandibular morphology assessment on OPT [13].

## 3. Results

**Figure 4:**
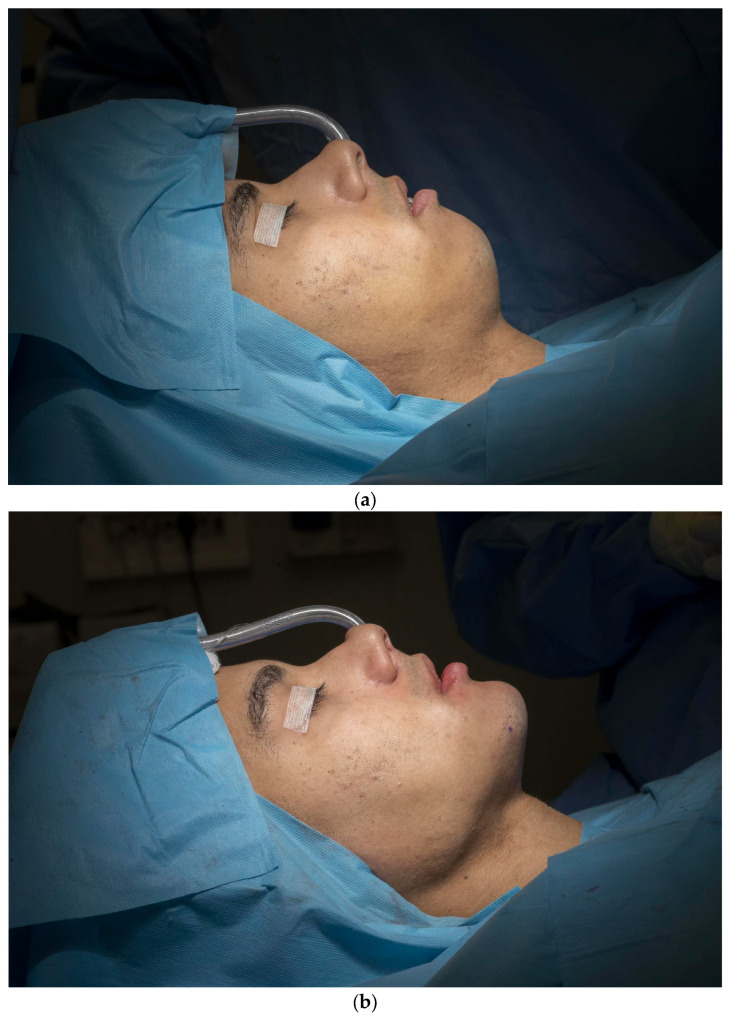
(**a**) Intraoperative presurgical aspect; (**b**) intraoperative immediately postsurgical aspect.

Between 1 January and 1 March 2025, seven male patients (mean age: 27.4 years; range: 23–39) were referred to our orthognathic surgery clinic by their orthodontists for the treatment of Class II dentoskeletal malocclusion. None of the patients presented relevant comorbidities. Initial evaluation revealed facial disharmony and asymmetry attributable to underdevelopment of the lower third of the face, characterized by mandibular retrusion in all patients. One patient exhibited an anterior open bite and occlusal plane canting, while another showed only occlusal canting. The remaining five patients presented with sagittal discrepancies without vertical or transverse occlusal alterations. CBCT revealed a high mandibular plane, a hyperdivergent mandibular body, and a short ramus. The surgical planning led to the decision to perform bimaxillary osteotomies with mandibular advancement and counterclockwise rotation. Positioned along the mandibular angle, as aforementioned, the Implate ensured the stability of the osseous segments and resulted in a more defined profile (Figure 4).

**Figure 5:**
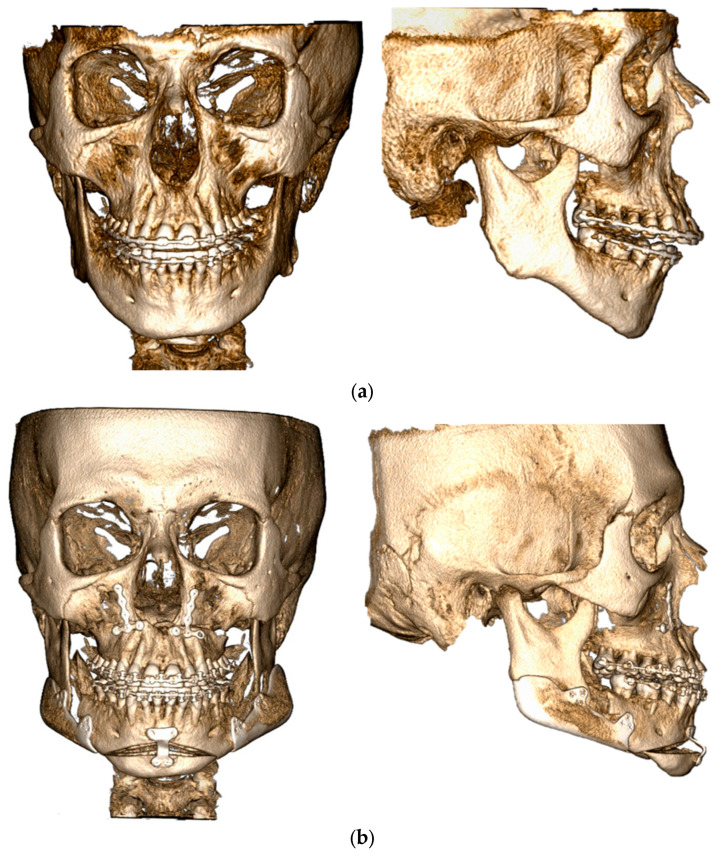
(**a**) Preoperative CT scan in frontal and lateral view; (**b**) one-month postoperative CT scan in frontal and lateral view.

The surgery produced stable and satisfactory outcomes, with proper occlusion and no significant postoperative complications, as confirmed by a CT scan performed one month after surgery (Figure 5).

Moreover, at the 6-month follow-up appointment and OPT analysis, the mean increase in mandibular width compared to preoperatively was 18.13 ± 6.16 mm, with values ranging from 9.3 to 25.8 mm. The mean increase in vertical ramus height was 6.03 ± 3.08 mm (range: 3.1–11.2 mm) on the left side and 5.76 ± 1.66 mm (range: 4.1–8.4 mm) on the right side (Table 1).

## 4. Discussion

The first mandibular osteotomy was performed in 1849 by Hullihen for the correction of an anterior open bite. Almost fifty years later, Whipple and Angle modified this procedure for the treatment of mandibular prognathism [14,15,16]. Subsequently, Blair introduced a new method for correcting maxillomandibular malocclusions, involving a blind transcutaneous osteotomy of the mandibular ramus. This technique was later refined by Kostecka through horizontal osteotomy [17,18]. These pioneering contributions marked a significant evolution in orthognathic surgery, enabling the treatment of various mandibular deformities. However, osteotomy techniques currently in use stem from subsequent methodological developments. In particular, the introduction of the intraoral sagittal split osteotomy by Trauner and Obwegeser represented a turning point, fostering further advances that improved the safety and precision of procedures [19]. These innovations allowed for greater accuracy in correcting mandibular anomalies and reducing intraoperative risks, expanding treatment possibilities and improving clinical outcomes for patients. The osteotomy proposed by Obwegeser aimed to reduce relapse rates and refine surgical outcomes. Over the years, the technique has been subject to continuous modifications by various surgeons, introducing measures aimed at optimizing bone contact, reducing the risk of nerve injury, and improving the aesthetic result. Today, variants of this technique are commonly known as bilateral sagittal split osteotomy (BSSO) and represent the standard in contemporary surgical practice. Building on this foundation, Hanna recently introduced Hanna’s Modified Sagittal Split Osteotomy (HSSO), an alternative to the BSSO for customized surgical interventions [20]. This method has been described as particularly suitable for treating facial asymmetries, such as mandibular asymmetry and hyperdivergent profiles with anterior open bite that require significant counterclockwise rotation of the mandible. The HSSO reflects growing attention paid to aesthetic aspects, which complements the traditional functional goal. While initial osteotomies primarily focused on functional outcomes, minimizing intraoperative complications, there is now a growing emphasis on facial aesthetic harmony, while maintaining the restoration of function as a priority. The HSSO offers a balance between aesthetic and functional improvements, allowing more precise manipulation of the mandibular angle, ramus height, and lower border, with a positive impact on facial profile harmony. However, despite the advances of modern osteotomy techniques, some limitations remain in meeting the demands of patients seeking simultaneous lowering of the mandibular ramus and widening of the mandibular angle. A key element in the stability of the surgery is the management of the mandibular condyle, which represents a complex challenge for orthognathic surgeons. Temporomandibular joint (TMJ) dysfunctions, relapses, and volumetric and positional alterations are common issues. Over the years, approaches aimed at preventing such complications have been developed, including condylar registration in the centric relation (CR) position and accurate repositioning of the mandibular rami through 3D digital planning software. Other key factors include the choice of fixation technique based on the movements of the anterior mandibular segment and proper management of muscle detachment. Extended skeletal stabilization with controlled management of the proximal mandibular segment is also a practical method available to achieve uniformly predictable stability in mandibular advancement surgery. To improve skeletal stability, orthognathic surgery today relies on advanced methodologies, such as controlled management of the proximal mandibular segment, allowing for more predictable surgical outcomes. Postoperative surgical relapse (SR) is often attributed to excessive tension in the soft and muscular tissues generated by mandibular advancement. This explains the increased frequency of SR in patients with a high mandibular plane angle (MPA), in whom the elongation of the masticatory muscles results in greater instability [21]. Another fundamental aspect concerns the preservation of the periosteum and minimization of periosteal and muscular detachment to maintain tissue integrity and reduce the risk of complications. The adoption of minimally invasive surgical techniques allows anatomical detachment to be limited, preserving the fibroelastic structure of the periosteum and ensuring better tissue adaptation to the implanted prosthesis for mandibular advancement. Minor detachment focused on the mandibular body rather than the ramus helps reduce unwanted movements of the condyle, improving overall mandibular stability. Particular attention must be paid to the management of the pterygomasseteric sling (PS), whose detachment can lead to retraction of the masseter muscle with consequent loss of soft tissue volume and a skeletal appearance of the mandibular angle [22]. This deformity can be prevented through careful subperiosteal dissection and reattachment of the muscle to the lower border of the mandible.

The use of appropriately sized implants fixed with screws reduces the risk of instability and the need for corrective interventions [7,23]. Based on these considerations, the Implate device represents an innovative solution for restoring the height of the mandibular ramus and increasing the transverse diameter of the angle, ensuring a harmonious and linear mandibular profile. Since there is a notable preference among the examined population for protrusive facial profiles over neutral profiles, emphasizing the importance of considering patients’ perspectives in the evolution of beauty standards, Implate offers innovative advantages in this context. This is in accordance with Rios et al.’s findings that the use of PSI in orthognathic surgery, combined with virtual planning, resulted in more precise correction of mandibular asymmetry, significant enhancement of jawline aesthetics, and high patient satisfaction, with a low rate of complications and no cases of implant exposure or significant infection during follow-up [24].

To our knowledge, no single technique currently allows the simultaneous correction of both the vertical height and transverse width of the mandibular ramus in a single procedure. These modifications must be addressed separately to achieve optimal results. Mandibular advancement and widening are further complicated by bone step formation, especially in Class II patients with shorter faces and hyperdivergent profiles, where vertical and transverse deficiencies at the mandibular angle are common. These steps, often radiographically detectable and palpable by patients, compromise the precision and aesthetics of the outcome. As a result, additional surgeries are frequently required to achieve a linear and harmonious mandibular profile. To date, no device has been developed that can address both vertical and transverse deficiencies simultaneously in a single intervention, underscoring the need to consider these as distinct surgical goals.

**Figure 6:**
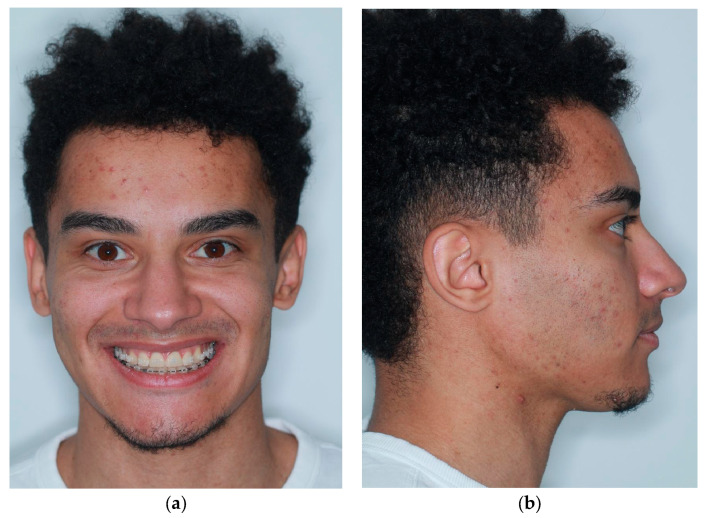
(**a**) Six months postoperative frontal view; (**b**) six months postoperative lateral view.

Implate provided essential stabilization while enhancing the facial profile by increasing the mandibular angle thickness (Figure 6).

Among short-term complications, prosthetic contamination by pathogens is almost always the most feared, affecting almost 5.3% of patients in a recent meta-analysis [25]. In cases where the proper antibiotic therapy does not allow the regression of the surgical site infection, a second surgery is necessary to remove the infectious source and resolve the issue [26]. During the development of Implate among the proposed designs, two options were evaluated: a porous prosthesis, which required a smaller amount of titanium, resulting in a lighter implant but with a larger potential surface area for pathogen colonization; or the solution we chose, a solid and uniform flat prosthesis, which, although slightly heavier, we consider to be safer, according to Gasik et al. [11].

**Figure 7:**
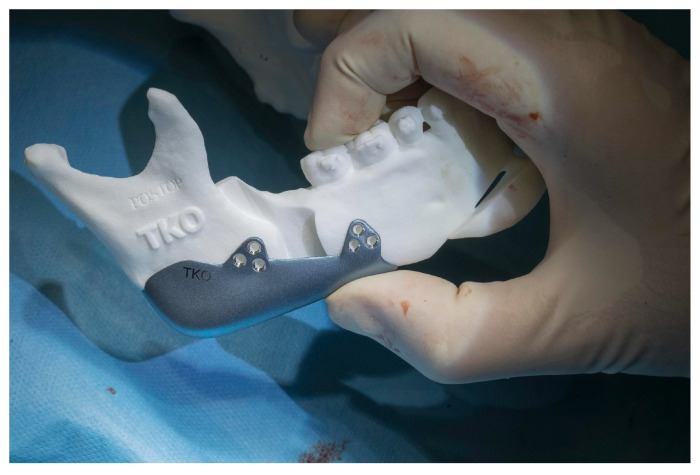
Implate positioning on anatomical mandibular patient reproduction.

Additionally, we believe that thanks to Implate, it is possible to ensure additional protection for the Inferior Alveolar Nerve (IAN). The incidence of numbness or altered nerve function after BSSO has been documented in a high percentage of operated sites [27,28]. Intraoperative damage to the IAN may occur through various mechanisms, and nerve function is probably more sensitive to compression than to tension. The method of fixation is a crucial factor influencing postoperative nerve function. The IAN can be injured during drilling or bicortical screw placement; instead, miniplate fixation caused significantly less nerve damage and patients recovered sensation more rapidly [29]. A meticulous preoperative radiographic assessment of the mandibular anatomy, along with a well-planned and executed operation, can help mitigate the risk of nerve damage. In this context, by carefully planning the positioning of the mandibular segments and thoroughly studying the patient’s anatomy, the insertion of Implate can be designed to avoid nerve compression while ensuring adequate bone contact for postoperative reossification. The main advantage of Implate over conventional miniplates lies in its ability to better adapt to the specific anatomical conformation of the patient (Figure 7). Although conventional miniplates are effective, they can exert undesirable pressure on the inferior alveolar nerve, increasing the risk of damage. Implate, thanks to its personalized and precise design, reduces this risk, making it safer in terms of nerve damage. Adequate bone contact also ensures that masticatory forces are evenly distributed, reducing the risk of postoperative complications such as insufficient osseointegration and subsequent mobilization of bone segments.

Despite the promising outcomes observed in this preliminary series, several limitations must be acknowledged. First, the small sample size (n = 7) restricts the generalizability of the results and prevents robust statistical analysis. All patients were male, further limiting the applicability of findings across diverse populations. Second, the study lacked a control group undergoing standard orthognathic surgery without the Implate device, which would have allowed for a more objective assessment of the device’s added value. Third, the follow-up period was relatively short (six months), and long-term data on bone remodeling, implant integration, and relapse rates are still lacking. Radiological and aesthetic outcomes were described qualitatively; future studies should employ validated quantitative metrics, Patient-Reported Outcome Measures, and blinded evaluators to reduce bias. Finally, the potential impact of the learning curve associated with the design and placement of PSI was not assessed. Further prospective studies with larger cohorts, control comparisons, and longer follow-up are necessary to confirm the efficacy, safety, and reproducibility of the Implate device in broader clinical settings.

## 5. Conclusions

In conclusion, integrating Implate into our surgical practice has significantly improved both functional and aesthetic outcomes in a pilot cohort of patients. While HSSO offers versatility in manipulating the mandibular structure, traditional osteotomy techniques alone cannot simultaneously address the vertical height and transverse diameter of the mandibular angle. Implate’s innovative design allows for simultaneous adjustments in both dimensions. This personalized approach, supported by meticulous preoperative surgical planning and advanced 3D printing technology, can also have the potential to minimize long-term complications and improve mandible stability. Larger prospective studies with control groups and extended follow-up are needed to confirm the efficacy and reproducibility of Implate in broader clinical settings.

## Figures and Tables

**Table 1:** Individual patient measurements of mandibular height and width before and after surgery.

Patient ID	Age	Left Height Pre- (mm)	Left Height Post- (mm)	Δ Left	Right Height Pre- (mm)	Right Height Post- (mm)	Δ Right	Width Pre-	Width Post-	Δ
1	26	41.7	48.5	6.8	41.8	47.5	5.7	96.3	118.1	21.8
2	26	54	63	9	53	60.7	7.7	96.5	105.8	9.3
3	27	63.2	66.3	3.1	59.7	68.1	8.4	93.4	116.7	23.3
4	39	43.2	47	3.8	46.5	50.9	4.4	96.1	114	17.9
5	25	43.2	54.4	11.2	49	53.8	4.8	104.6	122.4	17.8
6	23	50.3	54.3	4	45.3	49.4	4.1	99.4	125.2	25.8
7	26	51	55.3	4.3	50.5	55.7	5.2	99.3	110.3	11

## Data Availability

The data presented in this study are available on request from the corresponding author.
